# Synthesis, *in vitro* antitumour activity, and molecular docking study of novel 2-substituted mercapto-3-(3,4,5-trimethoxybenzyl)-4(3H)-quinazolinone analogues

**DOI:** 10.1080/14756366.2017.1368504

**Published:** 2017-09-26

**Authors:** Adel S. El-Azab, Alaa A.-M. Abdel-Aziz, Hazem A. Ghabbour, Manal A. Al-Gendy

**Affiliations:** a Department of Pharmaceutical Chemistry, College of Pharmacy, King Saud University, Riyadh, Saudi Arabia;; b Department of Organic Chemistry, Faculty of Pharmacy, Al-Azhar University, Cairo, Egypt;; c Department of Medicinal Chemistry, Faculty of Pharmacy, University of Mansoura, Mansoura, Egypt

**Keywords:** Quinazoline, *in vitro* antitumour evaluation, EGFR, molecular docking

## Abstract

A novel series of 2-substituted mercapto-3-(3,4,5-trimethoxybenzyl)-4(3H)-quinazolinones **1**–**20** was synthesised and evaluated for *in vitro* antitumour activity. *N*-(4-Chlorophenyl)-2-[(3-(3,4,5-trimethoxybenzyl)-4(3H)-quinazolinon-2-yl)thio)acetamide **(7)** and *N*-(3,4,5 trimethoxybenzyl)-2-[(3-(3,4,5-trimethoxybenzyl)-4(3H)-quinazolinon-2-yl)thio]propanamide **(19)** exhibited excellent antitumour properties, with mean growth inhibitory concentration (GI_50_) of 17.90 and 6.33 µΜ, respectively, compared with those of 5-fluorouracil 5-FU, gefitinib, and erlotinib (mean GI_50_: 18.60, 3.24, and 7.29 µΜ, respectively). Comparison of the GI_50_ (µM) values of compounds **7** and **19** versus those of 5-FU, gefitinib, and erlotinib against an *in vitro* subpanel of tumour cells lines showed that compounds **7** and **19** have activities almost equal to or higher than that of those standard drugs, especially against lung, CNS, and breast cancer cells. However, compounds **5**, **10**, **14**, **15**, **16**, **17,** and **20** exhibited effective antitumour activity against the different cell lines tested, with growth inhibition percentage (MGI%) of 19, 24, 19, 17, 16, 15, and 16, respectively. A modelling study was performed for compounds **7** and **19** by docking them into the EGFR kinase enzyme to study their mode of binding with the putative binding site.

## Introduction

**Figure 1.**
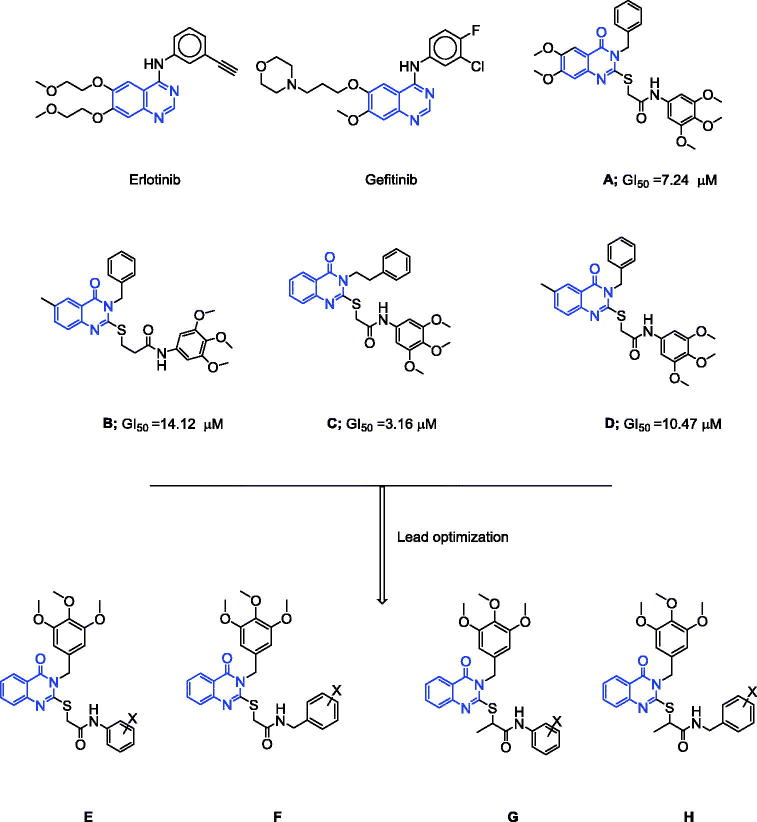
Structures of erlotinib, gefitinib, reported compounds **A**–**D**, and designed quinazoline derivatives **E**–**H** as antitumour agents.

Cancer refers to an abnormal growth of cells, and is the second leading cause of death worldwide^1^. Several of the current therapeutic agents have numerous side effects caused by their nonselective activity; therefore, the synthesis of safe and selective agents with a high therapeutic index is a vital research area. Quinazolinone nucleus is a characteristic bioactive scaffold present in several critical agents of biological interest^2–29^. Gefitinib and erlotinib (Figure 1) are known to contain a quinazoline nucleus and are effective in the treatment of breast and non-small cell lung (NSL) cancer via inhibition of epidermal growth factor receptor-tyrosine kinase (EGFR-TK)^30^^,^^31^. EGFR is over-expressed in numerous human tumours such as prostate, ovarian, breast, colon, and renal^31–34^. In our previously published studies^10^^,^^11^^,^^15^^,^^18^^,^^19^, the 2-mercaptoquinazoline analogue containing trimethoxyphenyl moiety showed significant antitumour activity such as 2-[(3-benzyl-6,7-dimethoxy-4(3H)-quinazolinon-2-yl)thio]-*N*-(3,4,5-trimethoxyphenyl)acetamide (**A**; GI_50_ = 7.24 µM), 2-[(3-benzyl-6-methyl-4(3H)-quinazolinon-2-yl)thio]-*N*-(3,4,5-trimethoxyphenyl)acetamide (**B**; GI_50_ = 14.12 µM), 2-[(3-phenethyl-4(3H)-quinazolinon-2-yl)thio]-*N*-(3,4,5-trimethoxyphenyl)acetamide (**C**; GI_50_ = 3.16 µM), 3-[(3-benzyl-6-methyl-4(3H)-quinazolinon-2-yl)thio]-*N*-(3,4,5-trimethoxyphenyl) propanamide (**D**; GI_50 _=14.12 µM) compared with that of the reference drug 5-fluorouracil (FU; mean GI_50_ 18.60 µM; Figure 1). In this study, we designed several new 2-substituted mercapto-3-(3,4,5-trimethoxybenzyl)quinazolin-4(3H)-ones containing various alkyl, acetamide, and isopropanamide fragments at position 2 of the quinazoline core, with different electronic environments that would affect lipophilicity. The synthesised molecules **2**–**20** were evaluated for their *in vitro* antitumour activities at a single dose (10 µM; Figure 1). These hybrids were synthesised with an aim to develop effective and selective antitumour molecules.

## Experimental

### Chemistry

Melting points were recorded on a Barnstead 9100 electrothermal melting apparatus. IR spectra (KBr) were recorded on an FT-IR Perkin-Elmer spectrometer (ν cm^−1^). ^1^H and ^13^C NMR spectra were recorded on Bruker 500 or 700 MHz spectrometers using DMSO-d_6_ as the solvent. Microanalytical data (C, H, and N) were obtained using a Perkin-Elmer 240 analyser and the proposed structures were within ±0.4% of the theoretical values. Mass spectra were recorded on a Varian TQ 320 GC/MS/MS mass spectrometer. Data of compound **8** were collected on a Bruker APEX-II D8 Venture area diffractometer (Billerica, MA), equipped with graphite monochromatic Mo *K*α radiation, *λ* = 0.71073 Å at 296 (2) K. Cell refinement and data reduction were carried out by Bruker SAINT. SHELXT^35^^,^^36^ was used to solve the structure.

### 2-Thioxo-3-(3,4,5-trimethoxybenzyl)-2,3-dihydroquinazolin-4(1H)-one (1)

A mixture of 3,4,5-trimethoxybenzyl isothiocyanate (11 mmol, 2.36 g), anthranilic acid (10 mmol, 1.37 g) and triethylamine (15 mmol, 1.51 g), was heated under reflux for 3 h in ethanol (20 ml). The reaction mixture was filtered while hot and the obtained solid was dried.

Yield: 86%; mp: 190–192 °C; IR (KBr, cm^−1^) *ν*: 1671 (C=O); ^1^H NMR (500 MHz, DMSO-d_6_): *δ* 13.04 (s, 1H), 7.98 (dd, 1H, *J* = 7.0 & 1.0 Hz), 7.77–7.70 (m, 1H), 7.42 (d, 1H, *J* = 8.0 Hz), 7.18 (*t*, 1H, *J* = 3.5 & 3.0 Hz), 6.86 (s, 2H), 3.72 (s, 6H), 3.61 (s, 3H); ^13^C NMR (125 MHz, DMSO-d_6_): *δ* 48.9, 55.8, 59.9, 105.2, 115.4, 115.6, 124.5, 127.3, 132.3, 135.5, 136.7, 139.0, 152.6, 159.4, and 175.6; Anal. calcd. for C_18_H_18_N_2_O4_S_ (%): C, 60.32; H, 5.06; N, 7.82. Found: C, 60.29; H, 5.08; N, 7.84; MS: [*m/z*, 358].

## General procedure for the synthesis of compounds 2–13

A mixture of 2-thioxo-3-(3,4,5-trimethoxybenzyl)-2,3-dihydroquinazolin-4(1H)-one **(1)** (1 mmol, 358 mg) and appropriate alkylhalides or 2-chloro-*N*-(substituted)acetamides (1 mmol) in 10 ml acetone containing potassium carbonate (2 mmol, 277 mg) was stirred at room temperature for 10–12 h. The reaction mixture was filtered, the solvent removed, and the obtained solid was washed with water and dried.

### 2-(Methylthio)-3-(3,4,5-trimethoxybenzyl)quinazolin-4(3H)-one (2)

Yield: 93%; mp: 174–175 °C; IR (KBr, cm^−1^) *ν*: 1670 (C=O); ^1^H NMR (500 MHz, DMSO-d_6_): *δ* 8.13 (dd, 1H, *J* = 6.5 & 1.5 Hz), 7.85–7.75 (m, 1H), 7.59 (d, 1H, *J* = 8.0 Hz), 7.50–7.44 (m, 1H), 6.60 (s, 2H), 5.26 (s, 2H), 3.70 (s, 6H), 3.63 (s, 3H), 2.62 (s, 3H); ^13^C NMR (125 MHz, DMSO-d_6_): *δ 14.7,* 47.0, 55.9, 59.9, 104.5, 118.6, 125.9, 125.9, 126.6, 131.3, 134.8, 136.9, 146.8, 152.9, 157.6, and 160.9; Anal. calcd. for C_19_H_20_N_2_O_4_S (%): C, 61.27; H, 5.41; N, 7.52. Found: C, 61.31; H, 5.39; N, 7.53; MS: [*m/z*, 372].

### 2-((2-Morpholinoethyl)thio)-3-(3,4,5-trimethoxybenzyl)quinazolin-4(3H)-one (3)

Yield: 88%; mp: 150–1152 °C; IR (KBr, cm^−1^) *ν*: 1683 (C=O); ^1^H NMR (500 MHz, CDCl_3_): *δ* 8.23 (d, 1H, *J* = 8.0 Hz), 7.69 (t, 1H, *J* = 7.5 Hz), 7.52 (d, 1H, *J* = 8.0 Hz), 7.38 (t, 1H, *J* = 7.5 Hz), 6.66 (s, 2H), 5.30 (s, 2H), 3.81 (s, 6H), 3.80 (s, 3H), 3.72–3.70 (m, 4H), 3.46–3.43 (m, 2H), 2.75–2.72 (m, 2H), 2.55–2.49 (m, 4H); ^13^C NMR (125 MHz, CDCl_3_): *δ* 29.3, 40.6, 47.6, 53.5, 53.7, 56.1, 57.4, 60.7, 66.8, 66.9, 105.4, 106.5, 119.3, 125.7, 126.0, 127.1, 131.3, 134.4,137.6, 147.3, 152.9, 153.2, 156.5, and 161.9; MS: [*m/z*, 471].

### 2-((2-(Piperidin-1-yl)ethyl)thio)-3-(3,4,5-trimethoxybenzyl)quinazolin-4(3H)-one (4)

Yield: 89%; mp: 162–164 °C; IR (KBr, cm^−1^) *ν*: 1680 (C=O); ^1^H NMR (500 MHz, CDCl_3_): *δ* 8.18 (d, 1H, *J* = 7.0 Hz), 7.635 (d, 1H, *J* = 6.0Hz), 7.49 (d, 1H, *J* = 7.0 Hz), 7.33 (d, 1H, *J* = 6.0 Hz), 6.63 (s, 2H), 5.26 (s, 2H), 3.77 (s, 9H), 3.41 (s, 2H), 2.48 (s, 4H), 1.57 (s, 4H), 1.41 (s, 2H); ^13^C NMR (125 MHz, CDCl_3_): 60.7, 57.6, 56.1, 54.3, 47.6, 29.4, 25.7, 24.1, 119.2, 126.0, 125.6, 127.0, 131.3, 134.4, 137.7, 147.3, 152.8, 156.5, and 161.9; MS: [*m/z*, 469].

### 2-((4-Chlorobenzyl)thio)-3-(3,4,5-trimethoxybenzyl)quinazolin-4(3H)-one (5)

Yield: 91%; mp: 174–175 °C; IR (KBr, cm^−1^) *ν*:, 1671 (C=O); ^1^H NMR (500 MHz, CDCl_3_): *δ* 8.26 (dd, 1H, *J* = 7.0 & 1.0 Hz), 7.75 (t, 1H, *J* = 7.0 0 Hz), 7.63 (d, 1H, *J* = 8.0 Hz), 7.43–7.40 (m, 3H), 7.30 (s, 1H), 7.28 (d, 1H, *J* = 2.0 Hz), 6.62 (s, 2H), 5.29 (s, 2H), 4.53 (s, 2H), 3.83 (s, 3H), 3.78 (s, 6H); ^13^C NMR (125 MHz, CDCl_3_): *δ* 161.9, 155.7, 153.2, 147.2, 137.6, 135.4, 134.5, 133.4, 131.1, 130.6, 128.7, 127.2, 126.0, 125.9, 119.4, 105.1, 60.8, 56.1, 47.6, and 35.8; Anal. calcd. for C_25_H_23_ClN_2_O_4_S (%):C, 62.17; H, 7.34; N, 5.80. Found: C, 61.22; H, 7.38; N, 5.78. MS: [*m/z*, 482; M + 1, 483].

### 2-((4-Oxo-3-(3,4,5-trimethoxybenzyl)-3,4-dihydroquinazolin-2-yl)thio)acetamide (6)

Yield: 81%; mp: 238–239 °C; IR (KBr, cm^−1^) *ν*: 3404 (NH), 1675, 1651 (C=O); ^1^H NMR (500 MHz, DMSO-d_6_): 8.30 (s, 1H), 8.12 (s, 1H), 7.81 (d, 1H, *J* = 5.0 Hz), 7.69 (s, 1H), 7.55 (d, 1H, *J* = 5.0 Hz), 7.47 (d, 1H, *J* = 5.5 Hz), 7.240 (d, 1H, *J* = 2.5 Hz), 6.66 (d, 1H, *J* = 6.0 Hz), 5.28 (s, 2H), 4.01 (d, 1H, *J* = 8.5 Hz), 3.73 (s, 6H), 3.66 (s, 3H); ^13^C NMR (125 MHz, DMSO-d_6_): *δ* 168.5, 160.8, 156.6, 152.9, 146.7, 136.9, 134.7, 131.2, 126.6, 126.0, 118.7, 104.6, 59.9, 55.9, 47.2, and 35.7; MS: [*m/z*, 415].

### N-(4-Chlorophenyl)-2-((4-oxo-3-(3,4,5-trimethoxybenzyl)-3,4-dihydroquinazolin-2-yl)thio)acetamide (7)

Yield: 84%; mp: 250–252 °C; IR (KBr, cm^−1^) *ν*: 3295 (NH), 1677, 1655 (C=O); ^1^H NMR (500 MHz, DMSO-d_6_): *δ* 10.47 (s, 1H), 8.10 (dd, 1H, *J* = 7.0 & 1.0 Hz), 7.70–7.72 (m, 1H), 7.62 (d, 2H, *J* = 9.0 Hz), 7.48 (d, 1H, *J* = 8.0 Hz), 7.45–7.40 (m, 1H), 7.30 (d, 2H, *J* = 8.5 Hz), 6.67 (s, 2H), 5.28 (s, 2H), 4.20 (s, 2H), 3.74 (s, 6H), 3.66 (s, 3H); ^13^C NMR (125 MHz, DMSO-d_6_): *δ* 36.8, 47.2, 55.8, 59.9, 78.5, 78.8, 79.0, 104.7, 118.7, 120.5, 125.7, 125.8, 126.5, 127.0, 128.5, 131.0, 134.6, 137.0, 137.8, 146.6, 152.8, 156.3, 160.8, ανδ 165.6; Anal. calcd. for C_26_H_24_ClN_3_O_5_S (%): C, 59.37; H, 4.60; N, 7.99. Found: C, 59.32; H, 4.61; N, 7.80. MS: [*m/z*, 525, M + 1, 526].

### N-(4-Fluorophenyl)-2-((4-oxo-3-(3,4,5-trimethoxybenzyl)-3,4-dihydroquinazolin-2-yl)thio)acetamide (8)

Yield: 83%; mp: 253–255 °C; IR (KBr, cm^−1^) *ν*: 3246 (NH), 1677, 1654 (C=O); ^1^H NMR (500 MHz, CDCl_3_): *δ* 9.72 (s, 1H), 8.34 (s, 1H), 7.84–7.28 (m, 5H), 6.96 (d, 2H, *J* = 5.0 Hz), 6.66 (s, 2H), 5.34 (s, 2H), 4.03 (s, 2H), 3.82 (s, 6H), 3.81 (s, 3H); ^13^C NMR (125 MHz, CDCl_3_): *δ* 166.4, 161.4, 157.7, 153.4, 146.4, 138.0, 135.3, 133.9, 130.4, 127.9, 126.8, 125.0, 121.0, 115.8, 115.6, 105.5, 60.8, 56.2, 48.1, and 36.1; MS: [*m/z*, 509].

### N-(4-Methoxyphenyl)-2-((4-oxo-3-(3,4,5-trimethoxybenzyl)-3,4-dihydroquinazolin-2-yl)thio)acetamide (9)

Yield: 85%; mp: 210–211 °C; IR (KBr, cm^−1^) *ν*: 3260 (NH) 1682, 1662 (C=O); ^1^H NMR (500MHz, CDCl_3_): *δ* 9.52 (s, 1H), 8.32 (d, 1H, *J* = 7.0 Hz), 7.82 (s, 1H), 7.66 (d, 1H, *J* = 7.5 Hz), 7.51 (d, 1H, *J* = 6.5 Hz), 7.34 (d, 2H, *J* = 8.5 Hz), 6.80 (d, 2H, *J* = 8.5 Hz), 6.66 (s, 2H), 5.33 (s, 2H), 4.03 (s, 2H), 3.82 (s, 6H), 3.80 (s, 3H), 3.76 (s, 3H); ^13^C NMR (125 MHz, CDCl_3_): *δ* 166.1, 161.4, 157.6, 156.3, 153.4, 146.5, 137.9, 135.2, 131.0, 130.5, 127.8, 126.7, 125.1, 121.0, 119.5, 114.2, 105.4, 60.8, 56.2, 55.4, 48.1, and 36.1; MS: [*m/z*, 521].

### 2-((4-Oxo-3-(3,4,5-trimethoxybenzyl)-3,4-dihydroquinazolin-2-yl)thio)-N-(3,4,5-trimethoxyphenyl)acetamide (10)

Yield: 83%; mp: 230–231 °C; IR (KBr, cm^−1^) *ν*: 3335 (NH), 1681, 1652 (C=O); ^1^H NMR (500 MHz, DMSO-d_6_): *δ* 8.27 (s, 1H), 8.15–8.10 (m, 1H), 7.80–7.76 (m, 1H), 7.58–7.53 (m, 1H), 7.46–7.42 (m, 1H), 7.01 (d, 2H, *J* = 20.5 Hz), 6.67 (d, 2H, *J* = 20.5 Hz), 5.29 (d, 2H, *J* = 19.0 Hz), 4.19 (d, 2H, *J* = 20.5 Hz), 3.78–3.63 (m, 18H); ^13^C NMR (125 MHz, DMSO-d_6_): *δ* 36.8, 47.2, 55.6, 55.8, 59.9, 60.0, 78.5, 78.84, 9.1, 96.8, 104.8, 118.8, 125.8, 126.6, 131.1, 133.5, 134.6, 134.9, 137.0, 146.7, 152.6, 152.9, 156.3, 160.8, and 165.2; MS: [*m/z*, 581].

### 2-((4-Oxo-3-(3,4,5-trimethoxybenzyl)-3,4-dihydroquinazolin-2-yl)thio)-N-(4-sulfamoylbenzyl)acetamide (11)

Yield: 81%; mp: 288–290 °C; IR (KBr, cm^−1^) *ν*: 3327, 3236 (NH), 1693 (C=O); ^1^H NMR (500 MHz, DMSO-d_6_): *δ* 10.78 (s, 1H), 8.11–8.10 (m, 1H), 7.80–7.76 (m, 5H), 7.47–7.43 (m, 2H), 7.26 (s, 2H), 6.69 (s, 2H), 5.29 (s, 2H), 4.26 (s, 2H), 3.74 (s, 6H), 3.65 (s, 3H); ^13^C NMR (125 MHz, DMSO-d_6_): *δ* 36.9, 47.3, 55.9, 59.9, 104.7, 118.6, 118.7, 125.7, 126.1, 126.6, 126.7, 131.2, 134.8, 136.9, 138.4, 141.8, 146.6, 152.9, 156.5, 160.8, and 166.3; MS: [*m/z*, 570].

### 2-((4-Oxo-3-(3,4,5-trimethoxybenzyl)-3,4-dihydroquinazolin-2-yl)thio)-N-(3,4,5-trimethoxybenzyl)acetamide (12)

Yield: 84%; mp: 203–205 °C; IR (KBr, cm^−1^) *ν*: 3260 (NH), 1682, 1662 (C=O); ^1^H NMR (500 MHz, DMSO-d_6_): *δ* 8.72 (t, 1H, *J* = 7.5 & 0.5 Hz(, 8.10 (d, 1H, *J* = 8.0 Hz), 7.73 (t, 1H, *J* = 7.5 & 0.5 Hz), 7.48–7.44 (m, 2H), 6.65 (s, 2H), 6.55 (s, 2H), 5.28 (s, 2H), 4.25 (d, 2H, *J* = 6.0 Hz), 4.09 (s, 2H), 3.71 (s, 6H), 3.64 (s, 6H), 3.62 (s, 3H), 3.61 (s, 3H); ^13^C NMR (125 MHz, DMSO-d_6_): δ 35.7, 42.9, 47.1, 55.6, 55.8, 59.9, 104.6, 104.7, 118.6, 125.9, 126.0, 126.5, 131.2, 134.7, 136.4, 136.9, 146.6, 152.7, 152.9, 156.5, 160.8, ανδ 166.7; Anal. calcd. for C_30_H_33_N_3_O_8_S (%):C, 60.49; H, 5.58; N, 7.05. Found: C, 60.51; H, 5.60; N, 7.03; MS: [*m/z*, 595].

### 2-((4-Oxo-3-(3,4,5-trimethoxybenzyl)-3,4-dihydroquinazolin-2-yl)thio)-N-(4-sulfamoylbenzyl)propanamide (13)

Yield: 81%; mp: 278–280 °C; IR (KBr, cm^−1^) *ν*: 3308, 3200 (NH), 1676, 1656 (C=O); ^1^H NMR (500 MHz, DMSO-d_6_): *δ* 8.86 (s, 1H), 8.13 (d, 1H, *J* = 7.0 Hz), 7.82–7.29 (m, 9H), 6.65 (s, 2H), 5.29 (s, 2H), 4.40 (s, 2H), 4.04 (d, 2H, *J* = 4.0 Hz), 3.70 (s, 6H), 3.64 (s, 3H); ^13^C NMR (125 MHz, DMSO-d_6_): *δ* 35.6, 42.2, 47.2, 55.8, 59.9, 104.6, 118.7, 125.5, 125.9, 126.1, 126.6, 127.2, 131.2, 134.8, 136.9, 142.5, 143.2, 146.6, 152.9, 156.5, 160.1, and 166.9; MS: [*m/z*, 584].

## General procedure for the synthesis of compounds 14–20

A mixture of 2-thioxo-3-(3,4,5-trimethoxybenzyl)-2,3-dihydroquinazolin-4(1H)-one **(1)** (1 mmol, 358 mg) and appropriate 2-chloro-*N*-(substituted)propanamides (1 mmol) in 10 ml acetone containing potassium carbonate (2 mmol, 277 mg) was heated under reflux for 6–9 h. The reaction mixture was filtered while hot, the solvent was removed, and the obtained solid was washed with water and dried.

### N-(4-Chlorophenyl)-2-((4-oxo-3-(3,4,5-trimethoxybenzyl)-3,4-dihydroquinazolin-2-yl)thio)propanamide (14)

Yield: 83%; mp: 222–224 °C; IR (KBr, cm^−1^) *ν*: 3253 (NH), 1685, 1655 (C=O); ^1^H NMR (500 MHz, DMSO-d_6_): *δ* 10.56 (s, 1H), 8.11 (dd, 1H, *J* = 7.0 & 1.0 Hz), 7.80 (t, 1H, *J* = 7.0 Hz), 7.66 (d, 2H, *J* = 9.0 Hz), 7.54 (d, 1H, *J* = 8.5 Hz), 7.46 (t, 1H, *J* = 7.0 Hz), 7.37 (d, 2H, *J* = 9.0 Hz), 6.64 (s, 2H), 5.24 (s, 2H), 4.76 (q, 1H, *J* = 9.0 Hz), 3.71 (s, 6H), 3.63 (s, 3H), 1.62 (d, 3H, *J* = 7.5 Hz); ^13^C NMR (125 MHz, DMSO-d_6_): *δ* 17.3, 46.5, 47.2, 52.0, 55.8, 59.9, 104.6, 118.8, 120.7, 125.7, 126.1, 126.6, 127.0, 128.6, 131.1, 134.8, 136.9, 137.8, 146.7, 152.9, 156.1, 160.7, and 169.5; MS: [*m/z*, 539, M + 1, 540].

### N-(4-Fluorophenyl)-2-((4-oxo-3-(3,4,5-trimethoxybenzyl)-3,4-dihydroquinazolin-2-yl)thio)propanamide (15)

Yield: 82%; mp: 216–217 °C; IR (KBr, cm^−1^) *ν*: 3302 (NH), 1685, 1661 (C=O); ^1^H NMR (500 MHz, DMSO-d_6_): *δ* 10.50 (s, 1H), 8.10 (d, 1H, *J* = 9.0 Hz), 7.79 (d, 1H, *J* = 7.5 Hz), 7.65 (q, 2H, *J* = 5.0 & 4.0 Hz), 7.56 (d, 1H, *J* = 8.0 Hz), 7.46 (d, 1H, *J* = 7.5 Hz), 7.13 (t, 2H, *J* = 9.0 & 8.5 Hz), 6.65 (s, 2H), 5.25 (s, 2H), 4.76 (dd, 1H, *J* = 7.0 Hz), 3.71 (s, 6H), 3.64 (s, 3H), 1.63 (d, 3H, *J* = 7.0 Hz); [*m/z*, 523].

### N-(4-Methoxyphenyl)-2-((4-oxo-3-(3,4,5-trimethoxybenzyl)-3,4-dihydroquinazolin-2-yl)thio)propanamide (16)

Yield: 84%; mp: 202–203 °C; IR (KBr, cm^−1^) *ν*: 3275 (NH), 1683, 1654 (C=O); ^1^H NMR (500 MHz, DMSO-d_6_): *δ* 10.29 (s, 1H), 7.81 (s, 1H), 8.11 (s, 1H), 7.57.47 (m, 3H), 6.89 (d, 2H, *J* = 4.0 Hz), 6.65 (d, 2H, *J* = 5.0 Hz), 5.25 (s, 2H), 4.76 (dd, 1H, *J* = 6.5 & 4.5 Hz), 3.71–3.64 (m, 12H), 1.62 (d, 3H, *J* = 6.5 Hz); ^13^C NMR (125 MHz, DMSO-d_6_): *δ* 17.6, 46.5, 47.2, 55.1, 55.9, 59.9, 104.6, 113.9, 118.8, 120.7, 120.9, 125.8, 126.1, 126.6, 131.1, 131.9, 134.8, 137.0, 146.7, 152.9, 155.4, 156.2, 160.7, and 168.7; MS: [*m/z*, 535].

### 2-((4-Oxo-3-(3,4,5-trimethoxybenzyl)-3,4-dihydroquinazolin-2-yl)thio)-N-(3,4,5-trimethoxyphenyl)propanamide (17)

Yield: 83%; mp: 206–207 °C; IR (KBr, cm^−1^) *ν*: 3324 (NH), 1684, 1664(C=O); ^1^H NMR (500 MHz, CDCl_3_-DMSO-d_6_): *δ* 10.32 (s, 1H), 8.20 (s, 1H), 8.09 (d, 1H, *J* = 8.0 Hz), 7.76 (d, 1H, *J* = 8.0 Hz), 7.56 (d, 1H, *J* = 8.0 Hz), 7.42 (d, 1H, *J* = 8.0 Hz), 6.98 (s, 2H), 6.63 (s, 2H), 5.23 (s, 2H), 4.76 (dd, 1H, *J* = 7.5 Hz), 3.72 (s, 12H), 3.65 (s, 3H), 3.62 (s, 3H), 1.62 (d, 3H, *J* = 7.0 Hz); ^13^C NMR (125 MHz, CDCl_3_-DMSO-d_6_): *δ* 17.3, 46.4, 47.1, 55.5, 55.7, 59.8, 59.9, 96.8, 96.9, 104.6, 118.8, 125.7, 125.9, 126.5, 131.0, 133.5, 134.5, 134.8, 136.9, 146.7, 152.6, 152.8, 156.1, 158.3, 158.5, 160.7, and 169.0; MS: [*m/z*, 595].

### 2-((4-Oxo-3-(3,4,5-trimethoxybenzyl)-3,4-dihydroquinazolin-2-yl)thio)-N-(4-sulfamoylphenyl)propanamide (18)

Yield: 81%; mp: 218–220 °C; IR (KBr, cm^−1^) *ν*: 3360, 3297 (NH), 1687, 1664 (C=O); ^1^H NMR (500 MHz, DMSO-d_6_): *δ* 10.78 (s, 1H), 8.10 (d, 1H, *J* = 7.0 Hz), 7.87–7.78 (m, 5H), 7.52 (d, 1H, *J* = 8.5 Hz), 7.46 (t, 1H, *J* = 7.5 & 8.0 Hz), 7.27 (s, 2H), 6.65 (s, 2H), 5.24 (d, 2H, *J* = 5.5 Hz), 4.78 (d, 1H, *J* = 7.0 Hz), 3.72 (s, 6H), 3.65 (s, 3H), 1.63 (d, 3H, *J* = 7.5 Hz); ^13^C NMR (125 MHz, DMSO-d_6_): *δ* 17.1, 46.6, 47.2, 55.8, 59.9, 104.6, 118.7, 120.7, 125.7, 126.1, 126.4, 126.6, 126.7, 131.1, 134.8, 136.9, 138.6, 139.8, 146.6, 152.9, 156.1, 160.7, and 170.0; [*m/z*, 584].

### 2-((4-Oxo-3-(3,4,5-trimethoxybenzyl)-3,4-dihydroquinazolin-2-yl)thio)-N-(3,4,5-trimethoxybenzyl)propanamide (19)

Yield: 83%; mp: 262–264 °C; IR (KBr, cm^−1^) *ν*: 3237 (NH), 1684, 1663 (C=O); ^1^H NMR (700 MHz, DMSO-d_6_): *δ* 8.84–8.77 (m, 2Η), 8.11 (d, 0.5H, *J* = 5.5 Hz), 7.76 (t, 0.5H, *J* = 5.5 Hz), 7.51–7.45 (m, 1H), 6.62 (s, 1H), 6.59 (s, 2H), 6.53 (s, 1H), 5.24 (s, 1H), 4.73–4.70 (m, 0.4H), 4.60–4.57 (m, 0.6H), 4.32–4.20 (m, 3H), 3.75–3.60 (m, 18H), 1.59–1.57 (m, 3H); ^13^C NMR (175 MHz, DMSO-d_6_): *δ* 18.4, 21.7, 42.8, 43.1, 46.1, 47.6, 54.8, 55.9, 56.1, 56.2, 60.3, 60.4, 104.6, 104.7, 104.8, 119.2, 126.4, 126.6, 127.0, 131.7, 134.9, 135.1, 135.2, 136.7, 136.8, 137.3, 147.2, 153.2, 153.3, 153.4, 156.6, 161.3, 169.2, and 170.9; MS: [*m/z*, 609]. Anal. calcd. for C_31_H_35_N_3_O_8_S (%): C, 61.07; H, 5.79; N, 6.89.Found: C, 61.12; H, 5.81; N, 6.91.

### 2-((4-Oxo-3-(3,4,5-trimethoxybenzyl)-3,4-dihydroquinazolin-2-yl)thio)-N-(4-sulfamoylbenzyl)propanamide (20)

Yield: 81%; mp: 174–175 °C; IR (KBr, cm^−1^) *ν*: 3371, 3253 (NH), 1685, 1663 (C=O); ^1^H NMR (500 MHz, DMSO-d_6_): *δ* 8.93 (s, 1H), 8.11 (d, 1H, *J* = 1.0 Hz), 7.82–7.80 (m, 1H), 7.64 (d, 2H, *J* = 8.5 Hz), 7.55–7.49 (m, 2H), 7.38 (d, 2H, *J* = 8.5 Hz), 7.29 (s, 2H), 6.61 (s, 2H), 5.24 (s, 2H), 4.69 (d, 1H, *J* = 7.5 Hz), 4.40–4.35 (m, 2H), 3.68 (s, 6H), 3.63 (s, 3H), 1.57 (d, 3H, *J* = 7.5 Hz); ^13^C NMR (125 MHz, DMSO-d_6_): *δ* 17.8, 42.1, 45.6, 47.1, 55.8, 59.9, 104.4, 118.7, 125.5, 125.7, 125.9, 126.2, 126.5, 127.2, 127.3, 131.2, 134.8, 136.8, 142.5, 143.1, 146.7, 152.8, 156.1, 160.8, and 170.7; MS: [*m/z*, 598].

### X-ray crystallography

Data of compound **8** were collected on a Bruker APEX-II D8 Venture area diffractometer, equipped with graphite monochromatic Mo *K*α radiation, *λ* = 0.71073 Å at 296 (2) K. Cell refinement and data reduction were carried out by Bruker SAINT. SHELXT^35^^,^^36^ was used to solve the structure. The final refinement was carried out by full-matrix least-squares techniques with anisotropic thermal data for non-hydrogen atoms on *F*. CCDC 1534954 contains the supplementary crystallographic data for this compound and can be obtained free of charge from the Cambridge Crystallographic Data Centre via www.ccdc.cam.ac.uk/data_request/cif.

## Antitumour screening

The antitumour evaluation was performed in nearly 60 human tumour cell lines obtained from nine organs, according to the rules of the Drug Evaluation Branch, NCI, Bethesda, MD^37–41^.

## Docking methodology

All modelling experiments were conducted with MOE 2007.9 of the Chemical Computing Group Inc. (Montreal, Canada)^42^^,^^43^. The starting coordinates of the X-ray crystal structure of the EGFR enzyme in complex with erlotinib (pdb code 1M17) were obtained from the RCSB Protein Data Bank^44^.

## Results and discussion

### Chemistry

**Scheme 1.**
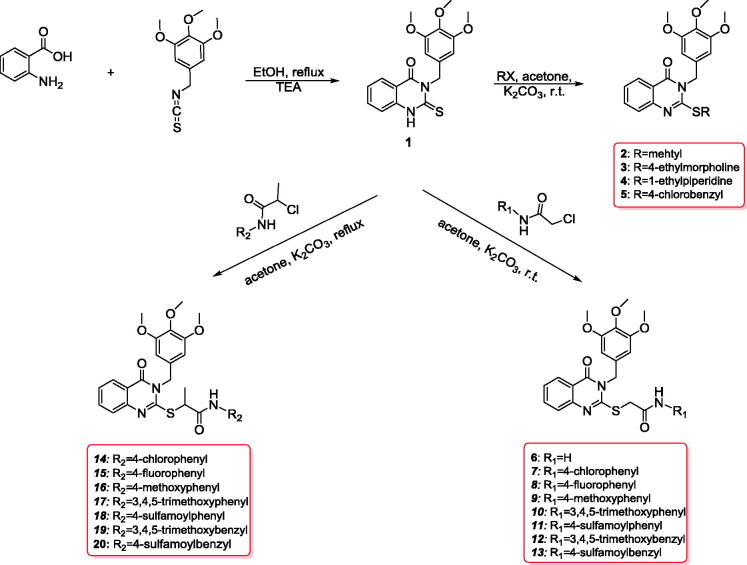
Synthesis of new quinazoline conjugates **1**–**20**.

2-thioxo-3-(3,4,5-trimethoxybenzyl)-2,3-dihydroquinazolin-4(1*H*)-one **(1)** was obtained at 86% yield by heating 2-aminobenzoic acid with 3,4,5-trimethoxybenzyl isothiocyanate in ethanol containing triethylamine (Scheme 1). The confirmation of compound **1** exists as thione tautomer in the solid-state according to X-ray of quinazoline analogue^45^^,^^46^ due to the dimeric aggregates are connected into layers by C=H···O interactions, involving the bifurcated carbonyl-O atom, and C—H···S interactions^45^^,^^46^.

The thione tautomer was confirmed by presence of singlet signal at 13.04ppm, corresponding to NH group and unique signal at 175.6 ppm related to C=S according to ^1^H NMR and ^13^C NMR spectra. Additionally, NMR spectra of compound **1** revealed three characteristic signals related to trimethoxybenzyl group at 59.9, 55.8, 48.9, 5.60, 3.72, and 3.68 ppm. Accordingly, compound **1** was stirred at room temperature with various halides (such as methyl iodide, 4-(2-chloroethyl)morpholine, 1-(2-chloroethyl)piperidine, and 4-chlorobenzylchloride) in acetone containing potassium carbonate to give 2-(substituted alkylthio)-3-(3,4,5-trimethoxybenzyl)quinazolin-4(3*H*)-ones **2**–**5** analogues at 88–93% yield (scheme 1). The ^1^H NMR spectra of compounds **2**–**5** showed loss of the NH group of the parent compound at 13.04 ppm, and a new signal related to s-alkyl moiety was observed at 4.61–2.62 ppm in the ^1^H NMR spectra and at 14.7–35.8 ppm in the ^13^C NMR spectra of these compounds.

Compound **1** was also stirred with various 2-chloro-*N*-(substituted)acetamides and 2-chloro-*N*-(substituted)propanamides in acetone containing potassium carbonate to give *N*-(substituted)-2-[(3-(3,4,5-trimethoxybenzyl)-4(3H)quinazolinon-2-yl)thio]acetamides **6**–**13** and *N*-(substituted)-2-[(3-(3,4,5-trimethoxybenzyl)-4(3H)quinazolinon-2-yl)thio]propanamides **14**–**20** at 81–86% yield (Scheme 1).

Compounds **6**–**13** were confirmed based on their ^1^H NMR spectra, which showed the presence of singlet signals at 10.78–8.30 ppm and 4.26–4.01 ppm attributable to –SCH_2_CONH– and –SCH_2_CONH– groups, respectively, in addition to characteristic signals of trimethoxybenzyl moieties at 5.34–5.28 ppm and 3.82–3.61 ppm. Similarly, ^13^C NMR spectra showed the presence of signals for –SCH_2_CONH– at 36.9–35.6 ppm and –SCH_2_CONH– groups at 168.5–165.2 ppm, accompanied by the characteristic signals of a trimethoxybenzyl moiety at 60.8–47.1 ppm and the carbonyl group of the parent quinazoline moiety at 161.4–160.1 ppm.

Based on the ^1^H NMR spectra, compounds **14**–**20** were recognised by the presence of signals for SCH_2_CONH– at 10.78–8.93 ppm, –SCH(CH_3_)CONH– groups at 4.78–4.69 ppm, and a typical peak for a SCH(CH_3_)CONH– moiety at 1.63–1.57 ppm, in addition to the classic signal of a trimethoxybenzyl moiety at 5.25–3.62 ppm. Simultaneously, these compounds were confirmed based on their ^13^C NMR spectra, which showed signals of –SCH(CH_3_)CONH–, –SCH(CH_3_)CONH–, and SCH(CH_3_)CONH– groups at 45.6–46.6, 17.1–17.8, and 169.0–170.8ppm, respectively, as well as the definitive signals of the trimethoxybenzyl and carbonyl groups of the parent quinazoline moiety at 47.1–59.9 and 160.6–160.8, respectively.

### X-ray crystallography

The crystallographic data and refinement information of compound **8** are summarised in Tables S1–S3. The asymmetric unit is comprised of one independent molecule as shown in Figures S1 and S2. All the bond lengths and angles are in normal ranges^47^. In the crystal structure, the central quinazolin-4(3H)-one plane makes dihedral angles of 62.97° and 68.48° with the trimethoxybenzyl and flurophenyl groups, respectively, in different directions. The crystal packing was formed by three intermolecular interactions between N_3_=H_1_N_3_•••O_2_, C_9_=H_9_A•••O_1_, and C_9_=H_9_B•••O_2_ with bond lengths 2.07 (3), 2.35, and 2.31 Å and bond angles 158(3)°, 143°, and 144°, respectively.

### Antitumour activity

Evaluation of the *in vitro* antitumour activity of the new synthesised compounds indicated in Table 1 was performed by the National Cancer Institute, Bethesda, MA. A single dose (10 µM) of the test compounds **2**–**20** was used in the full NCI 60 Human Tumor Cell Line Panel assay^37–41^.

**Table 1.** Percentage growth inhibition (GI %) of *in vitro* subpanel tumour cell lines at 10 µM concentration.

	% Growth Inhibition (GI %)																			
Subpanel tumour cell lines	2	3	4	5	6	7	8	9	10	11	12	13	14	15	16	17	18	19	20	5-FU
*Leukaemia*																				
CCRF-CEM	12	–	–	24	–	17	–	–	–	–	–	–	21	14	23	–	–	65	–	57.1
K-562	27	nt	13	38	–	55	14	21	–	21	nt	–	37	30	29	15	12	92	nt	42.3
MOLT-4	12	17	–	51	–	54	–	32	11	12	–	–	38	51	39	22	–	82	–	43.1
PRMI-8226	30	–	–	42	–	72	–	14	–	–	–	–	33	26	17	13	–	61	–	41.4
*SR*	17	–	–	44	–	54	–	18	34	12	–	–	39	44	24	37	20	89	–	24.8
Non-small cell lung cancer																				
A549/ATCC	–	–	–	15	–	36	–	–	38	–	–	–	19	17	15	14	14	65	14	34.2
HOP-62	35	13	–	11	–	46	36	–	58	–	–	16	15	18	16	21	–	71	64	47.8
NCI-H226	26	13	12	37	13	L	–	12	43	34	–	12	18	21	23	27	21	30	21	69.5
HOP-92	–	–	–	24	–	61	–	42	61	–	–	–	36	45	40	41	43	56	39	50.6
NCI-H23	–	–	–	15	11	28	38	–	23	–	–	–	13	–	11	–	–	49	–	39.0
NCI-H322M	–	–	–	–	–	36	–	–	18	–	–	–	28	19	13	11	–	40	–	59.5
NCI-H460	–	–	–		–	83	–	–	43	–	–	–	–	–	–	–	–	87	20	13.0
NCI-H522	29	22	32	49	11	60	27	44	67	30	19	17	51	47	40	32	30	90	62	58.0
Colon cancer																				
COLO 205	–	–	–	27	–	–	–	–	–	–	–	–	–	–	–	–	–	75	–	40.2
HCC-2998	–	nt	–	–	–	–	–	–	–	–	–	–	–	–	–	–	–	14	21	>100
HCT-116	18	–	–	44	–	65	–	11	42	14	–	–	44	35	31	16	24	84	–	17.8
HCT-15	–	–	–	31	–	28	–	11	–	–	–	–	27	21	24	12	–	83	–	26.5
HT29	–	–	–	41	–	13	–	–	–	–	–	–	13	14	–	–	–	88	–	27.1
KM12	–	–	–	–	–	21	–	–	–	–	–	–	–	–	–	–	–	83	–	40.7
SW-620	–	–	–	12	–	23	–	–	–	–	–	–	–	–	–	–	–	77	–	50.1
CNS cancer																				
SF-268	–	–	14	–	–	44	13	15	46	–	–	18	23	20	19	12	–	50	18	59.0
SF-295	23	–	–	–	–	38	–		15	–	–		16	12	–	–	–	64	–	69.1
SF-539	13	–	–	–	–	L	–	22	34	–	–	–	14	16	16	–	–	75	42	>100
SNB-19	–	–	–	19	–	37	–		26	16	–	–	21	20	23	18	17	45	33	65.9
SNB-75	22	15	27	34	12	73	52	34	L	27	11	21	26	20	36	34	36	83	77	65.9
U251	–	–	–	–	–	25	–	–	48	–	–	–	12	–	–	–	–	62	14	50.3
Melanoma																				
LOX IMVI	–	–	–	13	–	44	–	–	–	–	–	–	12	–	–	–	–	56	–	30.4
MALME-3M	–	–	–	–	–	91	–	14	18	12	–	–	17	–	–	17	13	60	13	58.2
M14	–	–	–	14	–	26	–	–	–	–	–	–	14	–	–	11	–	94	–	–
MDA-MB-435	–	–	–	–	–	31	–	–	–	–	–	–	–	–	–	–	–	L	–	36.6
SK-MEL-2	–	–	–	25	–	19	–	–	25	–	–	–	19	19	17	–	–	L	15	95.5
SK-MEL-28	–	–	–	–	–	17	12	–	–	–	–	–	–	–	–	13	–	45	–	–
SK-MEL-5	20	–	–	15	–	51	11	13	–	–	–	–	12	16	18	20	18	88	21	33.7
UACC-257	–	–	–	–	–	27	–	–	–	–	–	–	–	–	–	15	13	41	13	19.5
UACC-62	25	**–**	–	32	–	37	15	26	16	23	14	–	31	24	32	27	26	64	21	39.7
Ovarian cancer																				
IGROV1	–	–	–	24	–	59	–	–	28	11	–	–	14	23	23	22	24	49	–	51.2
OVCAR-4	34	–	30	37	–	47	28	18	11	15	–	–	23	15	30	26	23	42	12	59.4
OVCAR-5	–	–	–	–	–	18	–	–	12	–	–	–	–	–	–	–	–	32	–	44.3
OVCAR-8	–	–	15	15	–	38	20	–	20	–	–	13	15	16	14	–	14	52	–	–
NCI/ADR-RES	22	–	–	22	–	49	–	17	–	–	–	–	19	17	14	–	–	83	–	47.6
SK-OV-3	–	–	–	–	–	32	13	–	49	–	–	–	–	–	–	–	–	73	43	77.5
Renal cancer																				
786-0	–	–	–	–	–	L	–		27	–	–	–	11	–	–	–	–	44	–	48.7
A498	–	–	–	**–**	18	59	–	13	38	20	12	–	36	37	29	29	–	79	–	>100
ACHN	19	–	13	15	–	82	17	20	67	–	–	–	16	19	15	15	18	52	13	39.3
CAKI-1	25	–	–	22	–	29	–	–	–	–	–	–	29	26	14	11	–	66	–	39.4
RXF 393	–	–	–	–	–	81	22	18	39	–	–	–	23	26	22	26	–	47	34	34.3
SN12C	20	–	12	31	–	29	–	21	18	12	–	–	28	18	25	18	16	50	15	54.0
TK-10	–	–	–	0	–	33	16	–	33	–	–	–	–	–	–	–	–	38	–	66.9
UO-31	41	32	16	48	27	83	20	27	20	25	28	13	52	47	46	49	31	53	17	41.3
Prostate cancer																				
PC-3	18	–	–	41	13	35	–	15	13	–	–	–	28	25	25	17	19	51	–	58.2
DU-145	–	–	–	–	–	34	–	–	–	–	–	–	–	–	–		–	42	–	35.5
Breast cancer																				
MCF7	–	–	–	21	–	23	13	–	–	–	–	–	16	–	13	11	18	85	17	11.5
MDA-MB-231/ATCC	25	14	11	36	13	51	14	27	42	19	–	19	36	35	37	37	30	54	35	78.1
HS 578T	–	–	13		–	93	13	–	52	–	–	–	20	14	12	20	–	43	22	>100
BT-549	–	–	–		–	25	–	11	35	–	–	–	19	–	–	12	–	52	30	37.8
T-47D	14	–	15	36	–	55	–	16	23	–	11	–	15	23	32	24	–	91	36	56.7
MDA-MB-468	30	–	–	24	–	53	15	–	28	16	–	12	–	12	15	48	38	99	61	–
MGI%	11	2	4	19	2	47	7	10	24	7	2	1	19	17	16	15	10	65	16	
PCE	24/57	7/55	13/57	36/57	8/57	55/57	20/57	26/57	38/57	17/57	6/56	9/57	44/57	37/57	38/57	36/57	23/57	57/57	29/56	55/59

PCE: positive cytotoxic effect; the ratio between the number of cell lines with percentage growth inhibition >10% and total number of cell lines. MGI%: mean growth inhibition percentage; nt = not tested; L= >100%

The *in vitro* screening of compounds **2**–**20** at 10 µM showed that compounds **2, 4, 5, 7**–**11**, and **14**–**20** exhibited remarkable antitumour activities against the tested cell lines with positive cytotoxic effects (PCE) of 24/57, 13/57, 36/57, 55/57, 20/57, 26/57, 38/57, 17/57, 44/57, 37/57, 38/57, 36/57, 57/57, 23/57, and 29/56, respectively, compared with that of 5-FU (55/59) (Table 1). Conversely, compounds **3**, **6, 12**, and **13** showed weak activities against the tested cell lines with PCE of 7/55, 8/57, 6/56, and 9/57, respectively (Table 1).

2-(Substituted alkylthio)-3-(3,4,5-trimethoxybenzyl)quinazolin-4(3*H*)-ones **2**–**5** and 2-[(3-(3,4,5-trimethoxybenzyl)-4(3*H*)-quinazolinon-2-yl)thio]acetamide **(6)** showed variable antitumour activities with MGI % of 2–19 (Table 1).

*N*-(Substituted)-2-[(3-(3,4,5-trimethoxybenzyl)-4(3*H*)-quinazolinon-2-yl)thio]acetamides **7**–**13** showed mild to potent antitumour activities with MGI % ranging from 7 to 47, while *N*-(substituted)-2-[(3-(3,4,5-trimethoxybenzyl)-4(3H)quinazolinon-2-yl)thio]propanamides **14**–**20** showed potent antitumour activities with MGI % ranging from 10 to 65 (Table 1).

Compounds **3**, **4**, **6**, **8**, **11**, **12**, and **13** showed selective activity against different cancer cell lines. Compounds **3**, **4**, **11**, **12**, and **13** showed selective activity against the NCI-H522 cancer cell line, with a range of growth inhibition percentage (RGI %) of 17–32, while compounds **3**, **6**, **11**, and **12** had selectivity against the UO-31 cancer cell line with RGI % of 25–32. The SNB-75 cancer cell line was sensitive to compounds **4**, **11**, and **12** with RGI % of 21–27, whereas the MDA-MB-468 cancer cell line was sensitive to compounds **11** and **13** with RGI % of 16–19. The A498 cancer cell line was sensitive to compounds **6** and **11** with RGI % of 18–20, while the K-562, NCI-H226, UACC-62, and MDA-MB-231/ATCC cancer cell lines were susceptible to compound **11** with RGI % of 19–34. The MOLT-4, OVCAR-4, and SF-268 cancer cell lines were susceptible to compounds **3**, **4**, and **13** with RGI % of 17–30. The prostate cancer cell line PC-3 showed selective sensitivity to compounds **2**, **5**, **7**, and **14**–**18** with RGI % of 18–51; whereas compounds **7** and **19** showed selective activities with RGI % of 34–42 against the DU-145 prostate cell line (Table 1).

Furthermore, compounds **2**, **5**, **7**, **9**, **10**, and **14**–**20** showed potent activity against leukaemia, NSL cancer, colon cancer, CNS cancer, melanoma, ovarian cancer, renal cancer, and breast cancer cell lines with RGI % of 12–92, 16 –>100, 18–88, 13 –>100, 16 –>100, 16–83, 16 –>100, and 14–99, respectively (Table 1).

The MGI% data revealed that compounds **7** and **19** were the most active, with antitumour activity against numerous cell lines belonging to diverse tumour subpanels (Table 1). Therefore, these compounds were tested against a panel of 57 tumour cell lines at a 5-log dose range^37–41^ and the median growth inhibitory (GI_50_), total growth inhibitory (TGI), and median lethal (LC_50_) concentrations were calculated for each cell line (Table 2).

**Table 2.** Median growth inhibitory (GI_50_, μM), total growth inhibitory (TGI, μM), and median lethal (LC_50_, μM) concentrations of compounds **7** and **19** on *in vitro* subpanel tumour cell lines.

	Subpanel tumour cell lines										
Compd.	Activity	Leukaemia	NSC lung cancer	Colon cancer	CNS cancer	Melanoma	Ovarian cancer	Renal cancer	Prostate cancer	Breast cancer	MG-MID^a^
**7**	GI_50_	68.28	8.11	30.82	4.33	12.26	8.86	5.76	17.40	5.30	17.90
	TGI	^b^	40.56	85.81	15.28	57.06	37.30	30.41	^b^	30.46	55.20
	LC_50_	^b^	80.22	89.76	46.10	78.12	80.95	64.80	^b^	84.75	80.52
**19**	GI_50_	4.57	8.95	5.47	4.62	5.25	8.07	6.62	9.03	4.47	6.33
	TGI	93.44	63.45	73.35	63.85	49.74	56.50	65.06	^b^	70.50	70.65
	LC_50_	^b^	96.36	88.55	64.62	^b^	92.75	^b^	^b^	^b^	93.58
**5-FU**	GI_50_	15.10	^b^	8.40	72.10	70.60	61.40	45.60	22.70	76.40	18.60
	TGI	^b^	^b^	^b^	^b^	^b^	^b^	^b^	^b^	^b^	^b^
	LC_50_	^b^	^b^	^b^	^b^	^b^	^b^	^b^	^b^	^b^	^b^

a Full panel mean-graph midpoint (μM).

b Compounds showed values >100 μM.

Compounds **7** and **19**, compared with 5-FU, exhibited remarkable GI_50_ activities against leukaemia (68.28, 4.57, and 15.10 µM, respectively), NSL cancer (8.11, 8.95, and 100 µM), colon cancer (30.82, 5.47, and 8.40 µM), CNS cancer (4.33, 4.62, and 72.10 µM), melanoma cancer (12.26, 5.25, and 70.60 µM), ovarian cancer (8.86, 8.07, and 61.40 µM), renal cancer (5.76, 6.62, and 45.60 µM), prostate cancer (17.40, 9.03, and 22.70 µM), and breast cancer (5.30, 4.47, and 76.40 µM) (Table 2).

Additionally, comparing the median GI_50_ values (µM) of compounds **7** and **19** with those of 5-FU, gefitinib, and erlotinib against an *in vitro* subpanel of tumour cell lines showed that compounds **7** and **19** had activities almost equal to or higher than these known drugs against most cell lines (Table 3).

**Table 3.** GI_50_ values (μM) of compounds **7** and **19** compared with those of erlotinib, gefitinib, and 5-FU on *in vitro* subpanel tumour cell lines.

Subpanel tumour cell lines			GI50 (μM)		
	7	19	Erlotinib	Gefitinib	5-FU
Leukaemia					
CCRF-CEM	4.91	>100	15.84	5.01	31.62
HL-60(TB(	3.57	>100	5.01	5.01	19.95
MOLT-4	7.38	>100	5.01	3.98	12.58
RPMI-8226	4.00	22.90	5.01	1.58	5.01
SR	3.01	18.05	6.30	3.16	3.98
Non-small cell lung cancer					
A549/ATCC	6.33	4.66	7.94	7.94	1.99
HOP-62	4.36	3.10	12.58	10.00	19.95
HOP-92	5.70	2.26	6.30	7.94	>100
NCI-H226	14.60	4.00	6.30	15.84	>100
NCI-H23	6.87	16.5	19.95	15.84	12.58
NCI-H322M	23.00	19.1	0.05	0.08	19.95
NCI-H460	4.19	10.4	5.01	6.30	1.00
NCI-H522	6.55	4.89	1.00	6.30	39.81
Colon cancer					
COLO 205	5.32	73.20	31.62	6.30	nt
HCC-2998	13.00	26.60	79.34	10.00	nt
HCT-116	3.76	4.04	5.01	7.94	nt
HCT-15	2.47	17.00	3.16	5.01	nt
KM12	3.98	32.30	63.09	7.94	nt
SW-620	4.31	31.80	5.01	7.94	nt
CNS cancer					
SF-268	7.13	6.62	19.95	7.94	nt
SF-295	4.36	5.55	15.84	1.99	nt
SF-539	2.86	2.29	12.58	10.00	nt
SNB-19	6.00	6.80	3.98	12.58	nt
SNB-75	2.06	1.58	12.58	6.30	nt
U251	5.34	3.14	19.95	10.00	79.43
Melanoma					
LOX IMVI	7.05	13.50	5.01	7.94	6.30
M14	2.23	16.50	6.30	5.01	50.11
MDA-MB-435	1.15	22.80	15.84	3.16	10.00
SK-MEL-2	2.82	15.20	12.58	12.58	>100
SK-MEL-28	6.69	11.40	31.62	0.31	50.11
SK-MEL-5	2.85	6.38	15.84	3.98	12.58
UACC-257	16.30	6.04	100	6.30	>100
UACC-62	2.92	6.29	1.25	5.01	12.58
Ovarian cancer					
IGROV1	13.20	19.00	0.25	0.20	15.84
OVCAR-3	4.23	7.86	3.16	5.01	25.11
OVCAR-4	10.80	3.01	19.95	7.94	79.43
OVCAR-5	9.38	14.80	19.95	10.00	>100
OVCAR-8	10.00	4.82	7.94	10.00	19.95
NCI/ADR-RES	3.59	8.37	6.30	12.58	39.81
SK-OV-3	5.34	4.20	0.39	0.63	>100
Renal cancer					
786-0	5.10	3.37	5.01	7.94	12.58
A498	4.05	2.42	1.58	0.40	10.00
ACHN	8.09	3.15	0.15	0.20	10.00
CAKI-1	5.46	11.30	0.10	0.16	5.01
RXF 393	5.31	2.59	6.30	5.01	50.11
SN12C	8.10	16.90	6.3	6.30	25.11
TK-10	11.00	2.63	0.10	0.10	>100
UO-31	5.90	3.75	1.99	1.25	5.01
Prostate cancer					
PC-3	10.80	16.70	50.11	0.79	5.11
DU-145	7.27	18.10	1.58	2.51	50.11
Breast cancer					
MCF7	3.46	8.97	100	10.00	1.99
MDA-MB-231/ATCC	5.36	3.08	1.99	12.58	>100
HS 578T	5.24	3.95	6.30	10.00	>100
BT-549	4.90	6.42	39.81	7.94	100
T-47D	5.52	2.16	3.16	6.30	79.43
MDA-MB-468	2.35	7.22	0.20	0.01	31.62

nt: not tested.

### Structure-activity relationships

Structure activity relationships for antitumour activities with MGI % indicated that (i) 2-benzylmercapto-4(3*H*)-quinazolinone **5** showed higher antitumour activity (MGI%: 19%) than did the 2-alkylmercapto-4(3*H*)-quinazolinone derivatives such as compounds **2**–**4** (MGI%: 2–11%); (ii) *N*-(substituted phenyl)-2-[(3-(3,4,5-trimethoxybenzyl)-4(3H)-quinazolinon-2-yl)thio]acetamide analogues **7**–**11** (MGI%: 7–47%) and *N*-(substituted)-2-[(3-(3,4,5-trimethoxybenzyl)-4(3H)-quinazolinon-2-yl)thio]propanamide analogues **14**–**20** are more active than unsubstituted 2-[(3-(3,4,5-trimethoxybenzyl)-4(3*H*)-quinazolinon-2-yl)thio]acetamide **(6)**; (iii) the antitumour activity of *N*-(substituted)-2-[(3-(3,4,5-trimethoxybenzyl)-4(3H)-quinazolinon-2-yl)thio]propanamide analogues **14**–**20** is improved compared to that of *N*-(substituted)-2-[(3-(3,4,5-trimethoxybenzyl)-4(3H)-quinazolinon-2-yl)thio]acetamide analogues **6**–**13** except compounds **7** and **10**; (iv) the structure-activity correlation of *N*-(substituted)-2-[(3-(3,4,5-trimethoxybenzyl)-4(3*H*)-quinazolinon-2-yl)thio]acetamide analogues **6**–**13** revealed that *N*-(4-chlorophenyl)-2-[(3-(3,4,5-trimethoxybenzyl)-4(3H)-quinazolinon-2-yl)thio]acetamide **(7)** (MGI%; 47%) is more active than the corresponding *N*-(4-flourophenyl)acetamide **8** (MGI%; 7%); similarly, *N*-(3,4,5-trimethoxyphenyl)-2-[(3-(3,4,5-trimethoxybenzyl)-4(3H)-quinazolinon-2-yl)thio]acetamide **(10)** (MGI%; 24%) is more active than the corresponding *N*-(4-methoxyphenyl)acetamide **9** (MGI%; 10%). In addition, *N*-(4-methoxyphenyl)-2-[(3-(3,4,5-trimethoxybenzyl)-4(3*H*)-quinazolinon-2-yl)thio]acetamide **(9)** (MGI%: 10%) is more active than the corresponding *N*-(4-sulfamoylphenyl)acetamide **11** (MGI%: 7%); (v) The less active compounds in this series are *N*-(3,4,5-trimethoxybenzyl)-2-[(3-(3,4,5-trimethoxybenzyl)-4(3H)-quinazolinon-2-yl)thio]acetamide **(12)** (MGI%; 2%) and *N*-(4-sulfamoylbenzyl)-2-[(3-(3,4,5-trimethoxybenzyl)-4(3H)-quinazolinon-2-yl)thio]acetamide **(13)** (MGI%: 1%). Additionally, structure-activity correlation of *N*-(substituted)-2-[(3-(3,4,5-trimethoxybenzyl)-4(3H)-quinazolinon-2-yl)thio]propanamide analogues **14**–**20** indicates that: (i) *N*-(4-chlorophenyl)-2-[(3-(3,4,5-trimethoxybenzyl)-4(3H)-quinazolinon-2-yl)thio]propanamide **(14)** (MGI%: 19%) is more active than the corresponding *N*-(4-flourophenyl)propanamide **16** (MGI%: 17%); (ii) *N*-(3,4,5-trimethoxyphenyl)-2-[(3-(3,4,5-trimethoxybenzyl)-4(3*H*)-quinazolinon-2-yl)thio]propanamide **(17)** (MGI%: 15%) has the same antitumour activity as the corresponding *N*-(4-methoxyphenyl)propanamide **16** (MGI%: 16%); (iii) *N*-(4-methoxyphenyl)-2-[(3-(3,4,5-trimethoxybenzyl)-4(3*H*)-quinazolinon-2-yl)thio]propanamide **(16)** (MGI%: 16%) is more active than the corresponding *N*-(4-sulfamoylphenyl)propanamide **18** (MGI%: 10%); (iv) *N*-(3,4,5-trimethoxybenzyl)-2-[(3-(3,4,5-trimethoxybenzyl)-4(3*H*)-quinazolinon-2-yl)thio]propanamid **(19)** (MGI%: 65%) is more active than the corresponding *N*-(4-sulfamoylbenzyl)-2-[(3-(3,4,5-trimethoxybenzyl)-4(3H)-quinazolinon-2-yl)thio]propanamid **(20)** (MGI%: 16%).

### Molecular docking results

EGFR are tyrosine kinase enzymes that are overexpressed in numerous tumours such as colon, prostate, breast, ovarian, renal, and NSL cancers^31–34^^,^^48^. The inhibition of tyrosine kinase by quinazoline derivatives such as gefitinib and erlotinib (Figure 1) is well documented^30^^,^^31^. Accordingly, the antitumour activity of the target compounds against colon, prostate, breast, ovarian, renal, and NSL cancers encouraged us to study the molecular docking of the compounds into the putative binding site on EGFR kinase. In this study, the most active compounds **7** (mean GI_50_: 17.90 µΜ) and **19** (mean GI_50_:6.33 µΜ) were docked into the putative active site of EGFR kinase, as well as the reference inhibitor erlotinib (mean GI_50_: 7.29 µΜ)^44^. All docking calculations were performed using MOE 2007.09 software (MOE of Chemical Computing Group Inc., Montreal, Canada)^42^.

**Figure 2.**
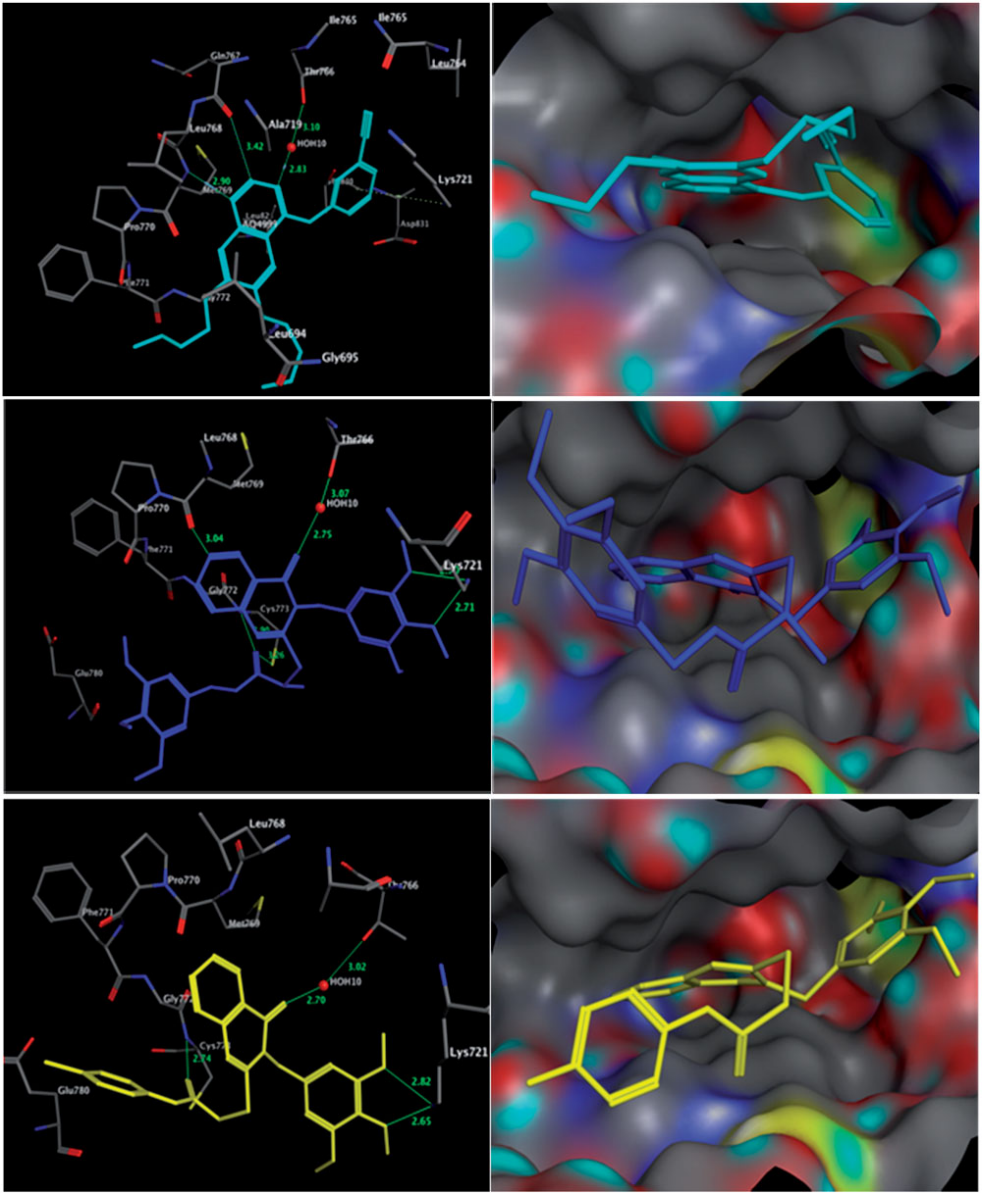
Three-dimensional (3D) interactions of erlotinib (upper panel), compounds **19** (middle panel) and **7** (lower panel) with the receptor pocket of EGFR kinase. Hydrogen bonds are shown with a green line.

The binding energies of the docked compounds **7**, **19**, and erlotinib (PDB code; 1M17)^44^ into the putative binding site of EGFR were −22.11, −25.21, and −26.99 kcal/mol, respectively (Figure 2). The molecular docking of the most active compound **19** revealed that it had similar orientation to erlotinib inside the receptor pocket, as well as additional bonding interactions. The docking results showed six typical and atypical hydrogen bonds with surrounding amino acids as shown in Figure 2. The trimethoxybenzyl fragment at C-3 of the quinazoline core formed bifurcated hydrogen bonds with amino acids Lys^721^. Moreover, the 4-quinazolinone ring uniquely formed two hydrogen bonds with the distinctive residues Met^769^ and Thr^766^, similar to that observed in erlotinib (Figure 2). Additionally, the carbonyl group of the acetanilide fragment of compound **19** formed bifurcated hydrogen bonds with the amino acid residue Cys^773^ and Gly^772^ augmenting the recognition within the enzyme binding site (Figure 2 and Table 4).

**Table 4.** Results of the docking of compounds **7** and **19** into EGFR (pdb: 1m17), in comparison to the co-crystallised ligand (erlotinib).

Ligand no.	No. of HBs^a^	Atoms in H-bonding in the ligand	Atoms in H-bonding in protein	Length^b^ (Å)
**7**	**4**	O of 3,4,5-timethoxyphenyl	NH of Lys^721^	2.65, 2.82
		O of quinazoline-4-one	HOH^10^ linked to Thr^766^	2.70
		O of carbonyl anilide	NH of Gly^772^	2.74
**19**	**6**	O of 3,4,5-timethoxyphenyl	NH of Lys^721^	2.70, 2.71
		O of quinazoline-4-one	HOH^10^ linked to Thr^766^	2.75
		O of carbonyl anilide	NH of Gly^772^	2.90
		O of carbonyl anilide	SH of Cys^773^	3.26
		Ar-H of quinazoline	O of Pro^770^	3.04
**Erlotinib**	**4**	N1 of quinazoline	NH of Met^769^	2.90
		N3 of quinazoline	HOH^10^ linked to Th^r766^	2.83
		Ar-H of quinazoline	NH of Leu^768^	3.42
		6-ring of anilino group	NH of Lys^721^	4.58

a HBs: hydrogen bonds;

b Length among acceptor and donner atoms in angstrom (Å).

Similar to compound **19**, compound **7** binds with four hydrogen bonds. It was found that the trimethoxybenzyl group at C-3 of the quinazoline core was clearly recognised with hydrogen bonding to the amino acid residue Lys^721^ similar to compound **19**, while the quinazoline core was shifted away from the distinctive amino acid residue Met^769^ (Figure 2). Additionally, two hydrogen bonds with the amino acid residue Gly^772^ and the distinctive residue Thr^766^ were found (Figure 2). It is obvious that the molecular docking results can be used to design novel quinazoline derivatives with potential binding to EGFR kinase and antitumour activity (Table 4).

## Conclusions

A novel series of 2-substituted mercapto-3-[3,4,5-trimethoxybenzyl]-4(3H)-quinazolinones **1**–**20**, was synthesised and evaluated for *in vitro* antitumour activity. Compounds **7** and **19** showed strong antitumour activities with mean GI_50_ values of 17.90 and 6.33 µM, TGI of 55.20 and 70.65 µM, and LC_50_ of 80.52 and 93.58 µM; these values were compared with the reference drug 5-FU (GI_50_: 22.60 µM, TGI: 100 µM, and LC_50_: 100 µM). Comparing the median GI_50_ (µM) of 5-FU, gefitinib, and erlotinib with that of compounds **7** and **19** showed that compounds **7** and **19** showed antitumour activities almost equal to or higher than that of the known drugs against most subpanel tumour cell lines. A molecular docking study for compounds **7** and **19** into the ATP binding site of EGFR-TK showed similar binding as that of erlotinib.

## Supplementary Material

IENZ_1368504_Supplementary_Material.pdf
